# UAV-Based Yield Prediction Based on LAI Estimation in Winter Wheat (*Triticum aestivum* L.) Under Different Nitrogen Fertilizer Types and Rates

**DOI:** 10.3390/plants14131986

**Published:** 2025-06-29

**Authors:** Jinjin Guo, Xiangtong Zeng, Qichang Ma, Yong Yuan, Nv Zhang, Zhizhao Lin, Pengzhou Yin, Hanran Yang, Xiaogang Liu, Fucang Zhang

**Affiliations:** 1 Yunnan Key Laboratory of Efficient Utilization and Intelligent Control of Agricultural Water Resources, Faculty of Modern Agricultural Engineering, Kunming University of Science and Technology, Kunming 650500, China; 20220127@kust.edu.cn (J.G.); 20242214075@stu.kust.edu.cn (X.Z.); maqichang@stu.kust.edu.cn (Q.M.); 20232214077@stu.kust.edu.cn (N.Z.); linzhizhao@stu.kust.edu.cn (Z.L.); yinpengzhou@stu.kust.edu.cn (P.Y.); yhr@stu.kust.edu.cn (H.Y.); 20090043@kust.edu.cn (X.L.); 2 Yunnan Intelligent Water-Fertilizer-Pesticide Integration Technology and Equipment Innovation Team, Faculty of Modern Agricultural Engineering, Kunming University of Science and Technology, Kunming 650500, China; 3 Yunnan International Joint Laboratory of Intelligent Agricultural Engineering Technology and Equipment, Faculty of Modern Agricultural Engineering, Kunming University of Science and Technology, Kunming 650500, China; 4 Seasonal Arid Region, Water-Soil-Crop System Observation and Research Station of Yunnan Province, Faculty of Modern Agricultural Engineering, Kunming University of Science and Technology, Kunming 650500, China; 5 Key Laboratory of Agricultural Soil and Water Engineering in Arid and Semiarid Areas of the Ministry of Education, Northwest A&F University, Xi’an 712100, China; zhangfc@nwsuaf.edu.cn

**Keywords:** winter wheat, N fertilization type and rate, leaf area index, remote sensing, yield prediction

## Abstract

The rapid and accurate prediction of crop yield and the construction of optimal yield prediction models are important for guiding field-scale agronomic management practices in precision agriculture. This study selected the leaf area index (LAI) of winter wheat (*Triticum aestivum* L.) at four different stages, and collected canopy spectral information and extracted vegetation indexes through unmanned aerial vehicle (UAV) multi-spectral sensors to establish the yield prediction model under the condition of slow-release nitrogen fertilizer and proposed optimal fertilization strategies for sustainable yield increase in wheat. The prediction results were evaluated using random forest (RF), support vector machine (SVM) and back propagation neural network (BPNN) methods to select the optimal spectral index and establish yield prediction models. The results showed that LAI has a significantly positive correlation with yield across four growth stages of winter wheat, and the correlation coefficient at the anthesis stage reached 0.96 in 2018–2019 and 0.83 in 2019–2020. Therefore, yield prediction for winter wheat could be achieved through a remote sensing estimation of LAI at the anthesis stage. Six vegetation indexes calculated from UAV-derived reflectance data were modeled against LAI, demonstrating that the red-edge vegetation index (CI_red edge_) achieved superior accuracy in estimating LAI for winter wheat yield prediction. RF, SVM and BPNN models were used to evaluate the accuracy and precision of CI_red edge_ in predicting yield, respectively. It was found that RF outperformed both SVM and BPNN in predicting yield accuracy. The CI_red edge_ of the anthesis stage was the best vegetation index and stage for estimating yield of winter wheat based on UAV remote sensing. Under different N application rates, both predicted and measured yields exhibited a consistent trend that followed the order of SRF (slow-release N fertilizer) > SRFU1 (mixed TU and SRF at a ratio of 2:8) > SRFU2 (mixed TU and SRF at a ratio of 3:7) > TU (traditional urea). The optimum N fertilizer rate and N fertilizer type for winter wheat in this study were 220 kg ha^−1^ and SRF, respectively. The results of this study will provide significant technical support for regional crop growth monitoring and yield prediction.

## 1. Introduction

Wheat (*Triticum aestivum* L.) is the most widely planted grain crop in the world and plays an important role in global food security and national economy [1]. Wheat is the staple food for 35% of the world’s population and is the primary source of energy and protein for humans [2]. Therefore, accurate and timely monitoring of wheat growth and prediction of crop yield before wheat harvest is essential for timely adjustment of field agronomic management measures and ensuring global food security [3]. The traditional yield measurement method is sampling surveys, which require a large area of destructive sampling and consume considerable time and labor costs [4]. In contrast, with the development of technology, remote sensing technology is widely used in grain yield prediction due to its advantages of good timeliness and low cost, and its ability to effectively cope with the problems of complex terrain, scattered cultivated land and diverse crops [5].

N fertilizer is one of the most important nutrient elements that limit the productivity of farmland ecosystems, contributing 30%~50% to the increase in grain production, especially in China’s agricultural production where it has played an irreplaceable role [6]. The Guanzhong Plain is the largest grain producing area in Shaanxi Province. However, the fertilization method in this region is extensive, and the phenomenon of excessive fertilization is very common, especially in the application of N fertilizer [7]. Chang et al. (2014) showed that the average N application rate of winter wheat in the Guanzhong region of Shaanxi was 152–265 kg ha^−1^, while the recommended N application rate was 120–226 kg ha^−1^ [8]. In addition, the most common fertilization mode of winter wheat in Guanzhong Plain is a one-time basal application of N fertilizer or topdressing urea at the jointing stage [9]. The soil nutrient supply under this fertilization mode is difficult to synchronize with crop nutrient demand, which not only greatly increases the risk of soil N leaching, but also causes negative effects, such as soil salinization and environmental pollution [10]. Previous studies have shown that the N release rate of slow-release N fertilizer as a new type of N fertilizer is synchronized with crop N uptake [11,12]. Slow-release N fertilizer can meet the N demand of crops while minimizing N loss, thereby promoting N uptake by crops and increasing crop yield. It is considered to be an effective way to optimize N fertilizer management [13]. Therefore, optimizing the application of N fertilizer can match the availability of soil N with crop demand throughout the growing season by changing the amount, type, placement and time of N fertilizer application. On the premise of ensuring grain yield, it can maximize crop yield and N use efficiency, and reduce N loss and environmental pollution as much as possible.

Leaf area index (LAI) is an important parameter that determines the nutritional status of crops so that monitoring LAI can provide a vital reference for field water and N management, crop growth performance, and yield estimation [14]. Previous studies have shown that modeling based on LAI may be a simpler and more rapid method than modeling based on whole plant dry matter mass, leaf dry matter mass, or stem dry matter mass [15]. In addition, N deficiency or surplus could significantly affect crop leaf growth [16]. Optimizing N management practices can delay leaf senescence, maintain the number of green leaves, produce more photosynthetic products, which are conducive to dry matter accumulation, thus increasing crop yield [17,18]. Conversely, monitoring the growth status of leaves during crop growth has important guiding significance for N fertilizer application [14,19]. However, traditional LAI measurement methods usually rely on destructive sampling in the field and manual measurement, which are time- and labor-intensive [20]. To solve this problem, remote sensing has been studied and recommended as an alternative method for non-destructive and real-time monitoring of crop status [21,22]. Unmanned aerial vehicle (UAV) remote sensing data have demonstrated significant potential in crop growth monitoring and yield prediction. Compared with field data collection, UAV remote sensing data provided a more convenient data collection process [23].

In recent years, remote sensing technology has been widely used in agricultural management and agricultural monitoring [24,25]. Previous studies have developed many vegetation indices to estimate LAI or crop growth status, and to guide N management in the field [14]. In addition, using remote sensing observation data can monitor crop growth in real time and objectively analyze crop growth status, thereby improving yield prediction results [26]. Previous studies demonstrated that the combination of agronomic variables and vegetation indices (VIs) can improve the monitoring accuracy of crop growth and yield [27]. Tilly et al. (2015) also showed that the fusion of plant height and hyperspectral vegetation index can improve the estimation accuracy of barley biomass [28]. Through plant height, canopy cover and VIs derived from UAV multi-spectral, good yield prediction accuracy can be obtained [29]. These results are based on the well-known fact that crop nutrient and yield status is reflected by the reflectance signature of leaves and plants. In addition, machine learning technology has also been used for crop yield prediction, because it can more easily incorporate auxiliary data into the prediction process by establishing linear and non-linear relationship models, thereby improving yield prediction [30,31]. VIs calculated from remote sensing images is used to correlate to yield variability through statistical and machine learning models [32]. Liu et al. (2017a) used multiple linear regression and neural network analysis to associate VIs with growth and physiological characteristics of winter wheat leaves [33]. Previous researchers combined VIs derived from UAV-based hyperspectral imagery and crop height using random forest regression (RFR) and partial least square regression (PLSR) models, and compared the accuracy of the two models in yield prediction, finding that the RFR model performed better than the PLSR model [24]. Using remote sensing technology combined with deep learning methods to establish models could well solve the problems of difficult data statistics, high manpower consumption and low accuracy in yield prediction [5].

Remote sensing has become a popular tool to monitor crop health, growth, measurement, and to determine the optimal time for harvesting and rapid near real-time crop yield estimation with minimal cost [34]. Many studies have been conducted on the effects of soil types, nutrients, crop management measures, varieties and meteorological conditions on crop production [4,24,35]. In addition, the effects of optimizing N fertilizer management on crop growth, yield and N use efficiency have been widely studied. However, integrating the UAV remote sensing platform with real-time and non-destructive monitoring of the growth status of winter wheat under slow-release N fertilization, accurately predicting the yield of winter wheat, and establishing quantitative models to evaluate fertilizer efficacy while developing optimized strategies for sustainable yield enhancement, remain critical research priorities requiring in-depth investigation. Therefore, the objectives of this study were to: (1) evaluate the potential of using vegetation indices derived from remote sensing images to estimate winter wheat yield under different N fertilizer types and rates; (2) choose more suitable remote sensing indices for predicting winter wheat yield under different N fertilizer types and N application rates; (3) identify the best timing and more accurate model to estimate winter wheat yield under different N fertilizer types and N application rates.

## 2. Materials and Methods

### 2.1. Experimental Site Description

**Figure 1:**
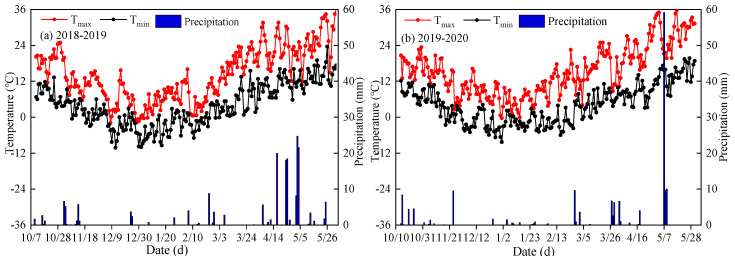
Daily temperature and precipitation during the winter wheat (*Triticum aestivum* L.) growing seasons of 2018–2019 (**a**) and 2019–2020 (**b**).

A two-season field experiment was carried out on winter wheat during October 2018–June 2019 and October 2019–June 2020 at the Northwest A&F University, Shaanxi Province, located in the southern Loess Plateau in northwest China (34°18′ N, 108°24′ E, 521 m a.s.l.). This area is a typical dryland agriculture region with annual average rainfall of 632 mm and potential evaporation of 1500 mm, respectively. Climatic variables were recorded during the two growing seasons of winter wheat and were monitored using an automated weather station (Figure 1). The soil texture of the 0–20 cm soil layer is silty clay loam, with soil pH of 8.14, dry bulk density of 1.40 g·cm^−3^, field capacity of 0.33 m^3^·m^−3^, total N of 0.93 g·kg^−1^, soil organic matter of 12.0 g·kg^−1^, available phosphorus of 21.42 mg·kg^−1^, and available potassium of 133.75 mg·kg^−1^, respectively.

### 2.2. Experimental Design and Measurements

Four N fertilizer types of traditional urea (TU), slow-release N fertilizer (SRF), mixed TU and SRF at a ratio of 2:8 (SRFU1), and mixed TU and SRF at a ratio of 3:7 (SRFU2) at four N fertilizer rates of 100 kg ha^−1^ (N1), 160 kg ha^−1^ (N2), 220 kg ha^−1^ (N3) and 280 kg ha^−1^ (N4). A control (CK) wheat grain field was set up with no N applied, and each treatment was repeated in three randomly distributed plots.

The sowing density of winter wheat (Xiaoyan 22 belongs to bread wheat) was 180 kg ha^−1^ with a row spacing of 20 cm. Each plot was designed with an area of 21 m^2^, containing 16 rows winter wheat. Winter wheat was seeded on 11 October 2018 and 14 October 2019, and harvested on 2 June 2019 and 31 May 2020. N fertilizers used in the experiments were urea (N ≥ 46%) and Shi Naian slow-release N fertilizer (N ≥ 25%, with a release period of three months, Fei Mu Ping Guo Commercial and Trading Co., Ltd., Linyi, China). The phosphate fertilizer was calcium superphosphate (P_2_O_5_ ≥ 16%, Yunnan Yuxi Chemical Fertilizer Factory Co., Ltd., Yuxi, China), and the potassium fertilizer was potassium sulfate (K_2_O ≥ 50%, Russia Import Trading Company, Moscow, Russia). For the TU treatment, 40% of the urea was applied before planting, while the remaining 60% was applied at the jointing stage (JO). The SRF, SRFU1 and SRFU2 treatment were applied as basal fertilizer, which were incorporated into the 0–15 cm soil layer before planting. The phosphate fertilizer (168 kg P_2_O_5_ ha^−1^) and potassium fertilizer (112 kg K_2_O ha^−1^) were also applied in each treatment before planting. Other field management practices followed local farmers’ customary methods. No obvious pests and diseases were observed in wheat during the experiment period.

### 2.3. Data Collaection

#### 2.3.1. UAV-Based Data Collection

In this study, the Micro-MCA system (Micro-MCA, Tetracam Inc., Chatsworth, CA, USA) was mounted on an UAV (M600, SZ DJI Technology Co., Ltd., Shenzhen, China) to obtain multi-spectral images of winter wheat canopy. Micro-MCA consisted of six individual miniature digital cameras for spectrum acquisition, the central wavelengths were 490 nm, 550 nm, 680 nm, 720 nm, 800 nm, 900 nm, and the width of each wavelength was 20 nm. The flights were carried out on 8 March, 31 March, 24 April, 13 May, during the 2018–2019 growing seasons of winter wheat, and 12 March, 7 April, 25 April, 13 May, during the 2019–2020 growing seasons of winter wheat. Flight operations were scheduled during four critical growth stages (when 70% of the crops stand exhibited diagnostic characteristics of each target phenological stage) of wheat. The UAV multi-spectral data was collected on clear and windless days between 11:00 and 14:00 AM local time, during which the change in solar zenith angle was minimal with sufficient radiation intensity. According to the pre-planned route, the flight altitude was kept at 50 m above the ground obtaining images at the spatial resolution of 2.5 cm, the camera shooting rate of 18–19 images per minute, and each test flight equipped with a whiteboard for image calibration.

The collected spectral images had been preprocessed by the software PixelWrench version 2, then processed images were imported into Pix4D software version 4.4.12 for image stitching and synthesis of multi-spectral tagged image file format (TIF) images of the experimental site. The method of vegetation index threshold [36] was used for the threshold segmentation of the preprocessed multi-spectral TIF images in the software ENVI version 5.3. After the soil background effects were removed based on the reflectance threshold between vegetation and background, the average reflectance of winter wheat canopy was obtained.

#### 2.3.2. Field Data Collection

Leaf area index: LAI was measured of each plot using the LI-COR LAI-2000 Plant Canopy Analyzer (Li-COR lnc., Lincoln, NW, USA) on 8 March (regreening stage), 31 March (jointing stage), 24 April (anthesis stage), 13 May (filling stage) during the 2018–2019 growing seasons of winter wheat, and 12 March (regreening stage), 7 April (jointing stage), 25 April (anthesis stage), 13 May (filling stage) during the 2019–2020 growing seasons of winter wheat. For each plot, the leaf area index was measured three times under consistent light direction and wheat row orientation, and the total number of samples was 153.

Grain yield: After winter wheat was harvested on 2 June 2019 and 31 May 2020, three 1 × 1 m^2^ of winter wheat grain was cut from each plot for destructive measurements of final yield, and the total number of samples was 153. The harvested winter wheat was air dried to a moisture content of 14% for seed threshed. All the dry seeds were weighted together, and then calculated and grain yield of each plot was reported.

### 2.4. Vegetation Indices Calculation

In this study, in order to investigate the influence of N types and rates, growth stages, and estimated winter wheat grain yield based on LAI, some VIs calculated from UAV-based images were utilized as shown in Table 1.

### 2.5. LAI Estimation Using Remotely Sensed Data

In this study, two techniques were tested for LAI estimation using canopy reflectance data. Firstly, the model between six vegetation indices and LAI of wheat in 2018–2019 was established by linear and non-linear regression analysis, and the spectral vegetation index with the strongest correlation with LAI was found (Figure S1). Then, the LAI observed in 2019–2020 was input into the model established above, and the simulated values and measured values of six vegetation indexes were analyzed to identify the spectral vegetation index with the best consistency between the simulated values and the measured values. Secondly, the noise equivalent of LAI was estimated by UAV image vegetation index (NDVI, OSAVI, EVI2, CI_red edge_, MTCI, NDRE). The noise equivalent (NEΔLAI) of each VI was calculated to evaluate the accuracy of estimating LAI by different VIs [43]. The noise equivalent is an indicator that measures the accuracy of VI response to LAI changes throughout its dynamic range. When the noise equivalent was lower, the accuracy of the evaluation was higher. The formulae of NEΔLAI are as follows:

(1) $$NE\Delta LAI=\frac{SE\{VI\;vs.\;LAI\}}{d(VI)/d(LAI)}$$

where SE{VI vs. LAI} is the standard error of the best fit function of this relationship, d(VI)/d(LAI) is the first derivative of the best fit function of the relationship VI vs. LAI.

### 2.6. Data Analysis

#### 2.6.1. Machine Learning Method

Taking CI_red edge_ as input variables, three machine learning methods of RF, SVM, and BPNN were used to model and predict the yield of wheat on MATLAB R2022a software. The ratio of the training set to the validation set was 2:1. The average value of the relevant results predicted by the machine learning model is the final model–fitting result in this experiment. In the construction of the RF model, the number of decision trees in the CI_red edge_ model and the yield model were both set to 500 after parameter optimization and multiple training. In this study, the kernel function type of the SVM model was set to “poly”, and the parameter penalty coefficients *C* and *γ* of the SVM model were optimized using the grid search method. According to the principle of minimum cross-validation error, *C* and *γ* were determined to be 20 and 0.02, respectively. The BPNN used in this study was provided by the Neural Network Toolbox in MATLAB. The transfer function of the hidden layer was set to TANSIG, and the Levenbeger–Marquardt (Train-LM) algorithm based on numerical optimization theory used as the network training function. The number of neurons in the middle layer directly affects the simulation performance of the network. Thus, after several trainings, we determined that the number of neurons in the middle layer was 15. Also, during training, the maximum number of iterations was set to 1000 and the training target was set to 1 × 10^−5^. After the neural network was trained, the test data were entered into the training network simulation to obtain the simulated values.

#### 2.6.2. Model Verification

The simulation accuracy was evaluated by coefficient of determination (R^2^), index of agreement (d), root mean square error (RMSE) and normalized root mean square error (nRMSE). R^2^ was a statistic used to measure the goodness of fit. When R^2^ was closer to 1, the simulation results were consistent with the measured values, indicating that the simulation accuracy was higher. EF was the Nash–Sutcliffe model efficiency coefficient, which is often used to quantify the prediction accuracy of simulation models; EF = 1 indicated that the simulation accuracy was higher. *d* was a descriptive measure, which was both a relative measure and bounded measure. For consistency, when d ≥ 0.9 was “excellent”; when 0.8 ≤ d < 0.9 was “good”; when 0.7 ≤ d < 0.8 was “moderate”; when d < 0.7 was “poor”. RMSE summarized the average difference between the simulated and observed values. nRMSE showed the relative size of the average difference without units. For consistency, when nRMSE < 10% was “excellent”; 10% ≤ nRMSE < 20% was “good”; 20% ≤ nRMSE < 30% was “moderate”; and nRMSE ≥ 30% was “poor”. The mathematical equations of each statistical metric are described as follows:

(2) $$R^{2}={\left[\frac{\sum_{i=1}^{n}\left(O_{i}-\bar{O})\times (S_{i}-\bar{S}\right)}{\sqrt{\sum_{i=1}^{n}{\left(O_{i}-\bar{O}\right)}^{2}\times \sum_{i=1}^{n}{\left(S_{i}-\bar{S}\right)}^{2}}}\right]}^{2}$$

(3) $$d=1-\left|\frac{\sum_{i=1}^{n}{\left(S_{i}-O_{i}\right)}^{2}}{\sum_{i=1}^{n}(\left|S_{i}-{\bar{O}}_{i}\right|+\left|O_{i}-{\bar{O}}_{i}\right|{)}^{2}}\right|$$

(4) $$EF=1-\frac{\sum_{i=1}^{n}{\left(S_{i}-O_{i}\right)}^{2}}{\sum_{i=1}^{n}{\left(O_{i}-\bar{O}\right)}^{2}}$$

(5) $$RMSE=\sqrt{\frac{1}{n}\sum_{i=1}^{n}{\left(S_{i}-O_{i}\right)}^{2}}$$

(6) $$nRMSE=\frac{RMSE}{{\bar{O}}_{i}}\times 100$$

(7) $$MRE=\frac{1}{n}\sum_{i=1}^{n}\frac{\left|S_{i}-O_{i}\right|}{O_{i}}\times 100$$

where: *S_i_* is the simulated value; $\bar{S}$ is the average of simulated values; *O_i_* is the observed value; ${\bar{O}}_{i}$ is the average of observed values; *n* is the total number of samples.

#### 2.6.3. Statistical Analysis

One-way analysis of variance (ANOVA) was conducted using SPSS software version 23. ANOVAs were conducted using N fertilizer types and rates as the primary effects and included two-way interaction. Multiple comparisons of mean annual values were performed using the least significant difference (LSD) at *p* < 0.05. Origin was used to create the figures. The algorithm for estimating LAI of winter wheat by CI_red edge_ was to establish an equation by using the measured LAI and CI_red edge_ value at the anthesis stage (the data were 17 treatments with 3 replicates).

## 3. Results

### 3.1. Grain Yield and LAI of Winter Wheat at Different Growth Stages

**Figure 2:**
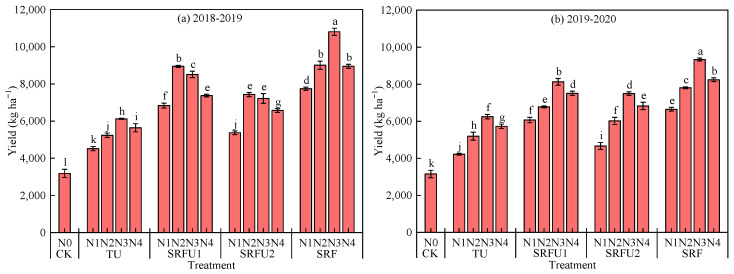
Measured yield of winter wheat under different nitrogen fertilizer types and nitrogen application rates in 2018–2019 (**a**) and 2019–2020 (**b**). TU is urea. SRFU1 and SRFU2 are urea blended with slow-release nitrogen fertilizer at ratios of 2:8 and 3:7, respectively. SRF is slow-release nitrogen fertilizer. Different letters indicate significance at the 5% level for the same year by the LSD test. Error bars are ±1 standard deviation of the mean (n = 3). The Y-bars on each data point should indicate SD.

Compared with CK treatment, N application could significantly increase grain yield of winter wheat (Figure 2). TU, SRFU1, SRFU2, SRF significantly increased grain yield by 68.79%, 148.38%, 108.51%, 186.41% in 2018–2019, and 69.51%, 125.76%, 98.15%, 153.82% in 2019–2020 compared with that in CK. Grain yield followed the order of SRF > SRFU1 > SRFU2 > TU in both 2018–2019 and 2019–2020. SRF increased grain yield by 69.69%, 15.31%, 37.36% in 2018–2019, and 49.74%, 12.43%, 28.10% in 2019–2020 compared with that in TU, SRFU1, and SRFU2. In 2018–2019, grain yield of TU and SRF reached the maximum values under N3, which were 8.6–35.6% and 20.7–39.5% higher than those in N1, N2 and N4, respectively. However, grain yield of SRFU1 and SRFU2 reached the maximum values under N2, which were 5.2–31.0% and 3.0–38.3% higher than those in N1, N3 and N4, respectively. In 2019–2020, grain yield of TU, SRFU1, SRFU2 and SRF reached the maximum values under N3, which were 9.0–48.0%, 8.2–33.9%, 9.8–60.6% and 13.3–40.5% higher than those in N1, N2 and N4, respectively. The maximum grain yield in SRFN3 was 10,805.29 kg ha^−1^ in 2018–2019 and 9334.04 kg ha^−1^ in 2019–2020, which were 19.93–239.06% and 13.28–195.88% in 2020 higher than those in other treatments, respectively.

LAI of TU increased first and then decreased during the whole growth period, and reached the maximum value at the jointing stage (Table 2). LAI of SRFU1, SRFU2 and SRF also increased first and then decreased during the whole growth period, but reached the maximum value at the anthesis stage. The LAI of winter wheat followed the order of SRF > SRFU1 > SRFU2 > TU over the two growing seasons. SRF increased maximum LAI by 42.2%, 12.9%, 14.3% in 2018–2019, and 8.7%, 4.2%, 7.2% in 2019–2020 compared with that in TU, SRFU1, SRFU2. Regarding the N fertilizer application rates, there was no significant difference in LAI of winter wheat under different N application rates at the regreening stage in 2018–2019 of winter wheat, the LAI of TU and SRF followed the order of N3 > N4 > N2 > N1, but the LAI of SRFU1 and SRFU2 followed the order of N2 > N3 > N2 > N1. During 2019–2020 growing seasons of winter wheat, the LAI of TU, SRFU1, SRFU2 and SRF followed the order of N3 > N4 > N2 > N1. NT, NR and NR×NT had highly significant effects on LAI during the two growing seasons of winter wheat (*p* < 0.01) (Table 2), except for that NT and NR × NT had no significant effects at the regreening stage (*p* > 0.05).

### 3.2. Yield Evaluation by LAI of Winter Wheat

#### 3.2.1. Yield Evaluation by LAI at Different Growth Stages

Correlation analysis between LAI at the regreening stage, jointing stage, anthesis stage and filling stage with grain yield of winter wheat were conducted (Table 3). The grain yield of winter wheat was significantly positively correlated with the LAI during the four growth stages (*p* < 0.01). During the anthesis stage and filling stages, the grain yield of winter wheat was closely related to LAI, and the Pearson coefficient was greater than 0.94 and 0.7 in 2018–2019 and in 2019–2020, respectively. However, the grain yield was weakly correlated with LAI at the regreening stage and jointing stages, with Pearson coefficients was 0.47 and 0.66 in 2018–2019, and 0.67 and 0.53 in 2019–2020, respectively. Over the two growing seasons of winter wheat, the highest Pearson correlation (0.96 in 2018–2019 and 0.83 in 2019–2020) between grain yield and LAI was obtained at anthesis stage, respectively. In conclusion, it is most appropriate to use LAI at the flowering stage to evaluate its potential yield over the whole growing season of winter wheat.

#### 3.2.2. Yield Estimation Based on LAI of Winter Wheat at the Anthesis Stage

**Figure 3:**
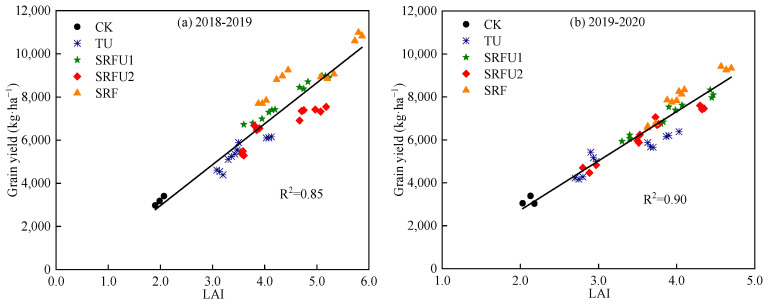
The relationship of grain yield and LAI at the anthesis stage in 2018–2019 and 2019–2020. (**a**) The relationship of grain yield and LAI at the anthesis stage in 2018–2019. (**b**) The relationship of grain yield and LAI at the anthesis stage in 2019–2020. CK is no N application. TU is urea. SRFU1 and SRFU2 are urea blended with slow-release nitrogen fertilizer at ratios of 2:8 and 3:7, respectively. SRF is slow-release nitrogen fertilizer.

**Figure 4:**
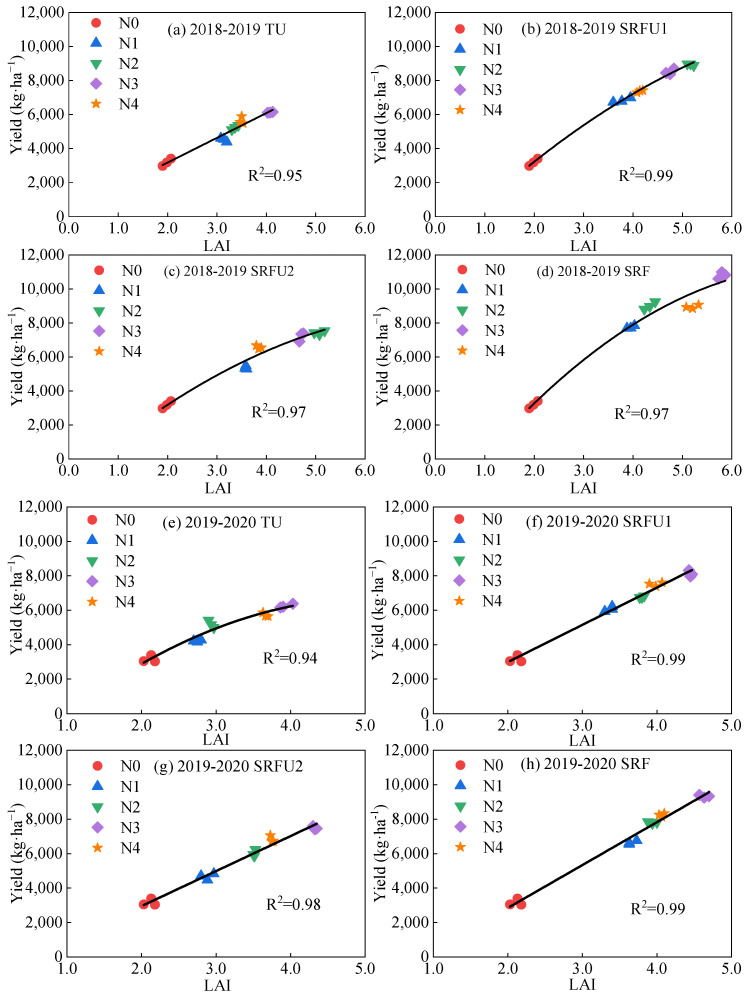
The relationship of yield and LAI at the anthesis stage of winter wheat under different nitrogen fertilizer types. (**a**,**e**) The relationship of yield and LAI at anthesis stage of winter wheat under TU in 2018–2019 and 2019–2020, respectively. (**b**,**f**) The relationship of yield and LAI at anthesis stage of winter wheat under SRFU1 in 2018–2019 and 2019–2020, respectively. (**c**,**g**) The relationship of yield and LAI at anthesis stage of winter wheat under SRFU2 in 2018–2019 and 2019–2020, respectively. (**d**,**h**) The relationship of yield and LAI at anthesis stage of winter wheat under SRF in 2018–2019 and 2019–2020, respectively. TU is urea. SRFU1 and SRFU2 are urea blended with slow-release nitrogen fertilizer at ratios of 2:8 and 3:7, respectively. SRF is slow-release nitrogen fertilizer.

Figure 3 shows the relationship between grain yield and LAI at the anthesis stage in 51 winter wheat plots. Overall, the LAI of all samples was significantly linearly correlated with grain yield, and the equations were Yield = −827.30 + 1896.03 LAI (R^2^ = 0.85) and Yield = −1858.49 + 2293.14 LAI (R^2^ = 0.90) in 2018–2019 and 2019–2020, respectively (Figure 3 and Table 4). Then, four N fertilizer types were carried out on 51 winter wheat plots, and the relationship between grain yield and LAI at the anthesis stage was established by using four N fertilizer types. When the samples were separated by N fertilizer types, the accuracy of LAI estimation yield was significantly improved (Figure 4). Regarding the various N fertilizer types, LAI was closely related to yield (R^2^ > 0.85), and there was no significant difference in the relationship between grain yield and LAI at the anthesis stage under various N treatments (Figure 4). In 2018–2019, grain yield and LAI at the anthesis stage showed a linear relationship under TU (Yield = 261.40 + 1455.88 LAI), and a non-linear relationship in SRFU1, SRFU2 and SRF (Yield = −1976.77 + 2901.34 LAI − 150.79 LAI^2^, Yield = −1268.48+2547.84 LAI–161.14 LAI^2^ and Yield = −3287.88 + 3762.64 LAI − 241.50 LAI^2^). However, there was a non-linear relationship between grain yield and LAI at the anthesis stage in TU (Yield = −3588.13 + 4016.09 LAI − 390.40 LAI^2^), and a linear relationship in SRFU1, SRFU2 and SRF (Yield = −1338.63 + 2163.98 LAI, Yield = −1116.09 + 2033.90 LAI and Yield = −2134.29 + 2489.85 LAI) in 2019–2020 (Table 4).

### 3.3. LAI at the Anthesis Stage Estimation Using Remotely Sensed Data

#### 3.3.1. LAI vs. Different Remotely Sensed Data Relationship for Winter Wheat

**Figure 5:**
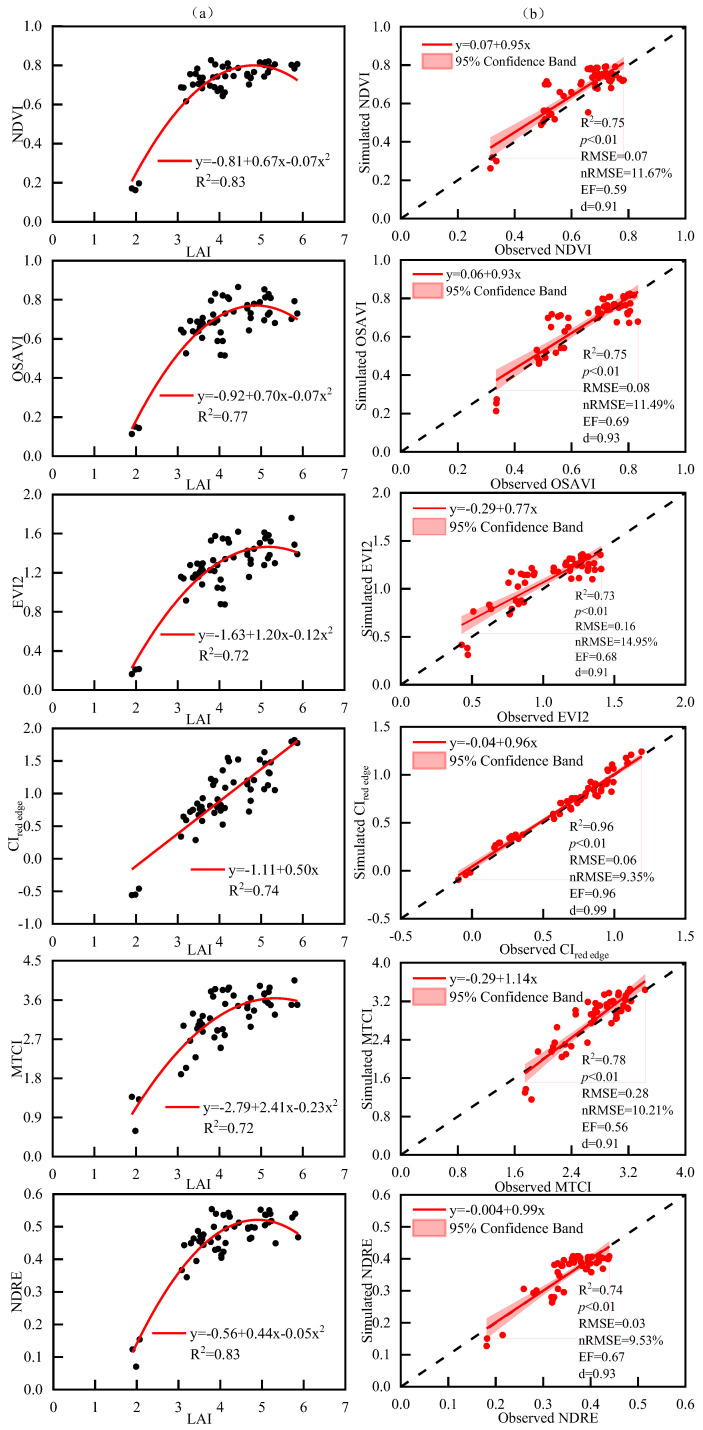
(**a**) The relationship of vegetation index (NDVI, OSAVI, EVI2, CI_red edge_, MTCI and NDRE) from UAV images and LAI of winter wheat in 2018–2019. (**b**) Comparison of simulated and observed vegetation index (NDVI, OSAVI, EVI2, CI_red edge_, MTCI and NDRE) of winter wheat in 2019–2020.

The above results determined that the LAI at the anthesis stage was used to estimate grain yield. Next, the LAI was estimated using remotely sensed data of the anthesis stage to achieve the goal of remote prediction of grain yield. The relationship between LAI and six widely used VIs retrieved from UAV platform were established (Figure 5). Based on the data of 51 plots in the 2018–2019 growing season, it was found that VIs were significantly positively correlated with LAI at the anthesis stage, having R^2^ ≥ 0.72 in all samples under different N fertilizer types and N application rates (Figure 5). Among them, NDVI, OSAVI, EVI2, MTCI and NDRE were non-linearly correlated with LAI (y = −0.81 + 0.67x − 0.07x^2^, y = −0.92 + 0.70x − 0.07x^2^, y = −1.63 + 1.20x − 0.12x^2^, y = −2.79 + 2.41x − 0.23x^2^ and y = −0.56 + 0.44x − 0.05x^2^) (Figure 5a), while CI_red edge_ was linearly correlated with LAI (y = −1.11 + 0.50x) (Figure 5a). The equation constructed by the VIs and the LAI in 2018–2019 was used to predict the VIs in 2019–2020, and then the simulated value of VIs was compared with the observed value in 2019–2020 (Figure 5b). The results showed that the equation constructed by VIs and LAI at the anthesis stage of winter wheat had good simulation results (R^2^ ≥ 0.74, nRMSE ≥ 9.35%, EF ≥ 0.56 and d ≥ 0.91) (Figure 5b). Among them, the simulated value of CI_red edge_ was the closest to the observed value, R^2^ = 0.96, nRMSE = 9.35%, EF = 0.96 and d = 0.99.

**Figure 6:**
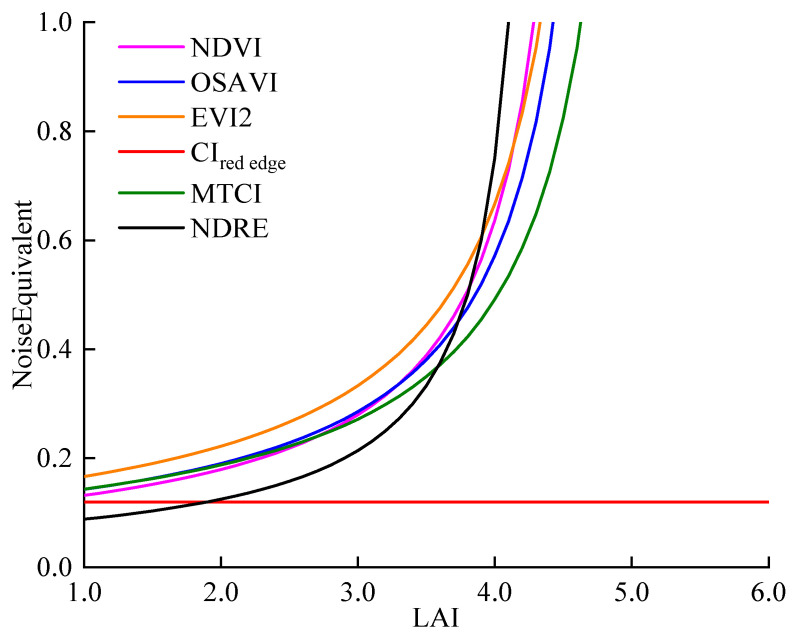
Noise equivalent of LAI estimation by vegetation index (NDVI, OSAVI, EVI2, CI_red edge_, MTCI and NDRE) from UAV images.

The noise equivalent values (NEΔLAI) of six VIs were calculated, and the dynamic response of NEΔLAI to LAI changes was analyzed (Figure 6). NDVI, EVI2 and NDRE could accurately estimate the low to moderate LAI with lower NEΔLAI, but their NEΔLAI sharply increased when LAI exceeded 2.7 m^2^/m^2^. In addition, the NEΔLAI of OSAVI and MTCI sharply increased when the LAI was greater than 2.9 m^2^/m^2^ and 3.1 m^2^/m^2^, respectively. However, the NEΔLAI of CI_red edge_ remained constant with the increase in LAI, and was smaller than the NEΔLAI of the other five VIs. Overall, the remote sensing data of CI_red edge_ had high accuracy and precision in responding to LAI changes during the whole dynamic range, and it could be used to evaluate the LAI of winter wheat.

#### 3.3.2. LAI vs. CI_rededge_ Relationship for Winter Wheat

**Figure 7:**
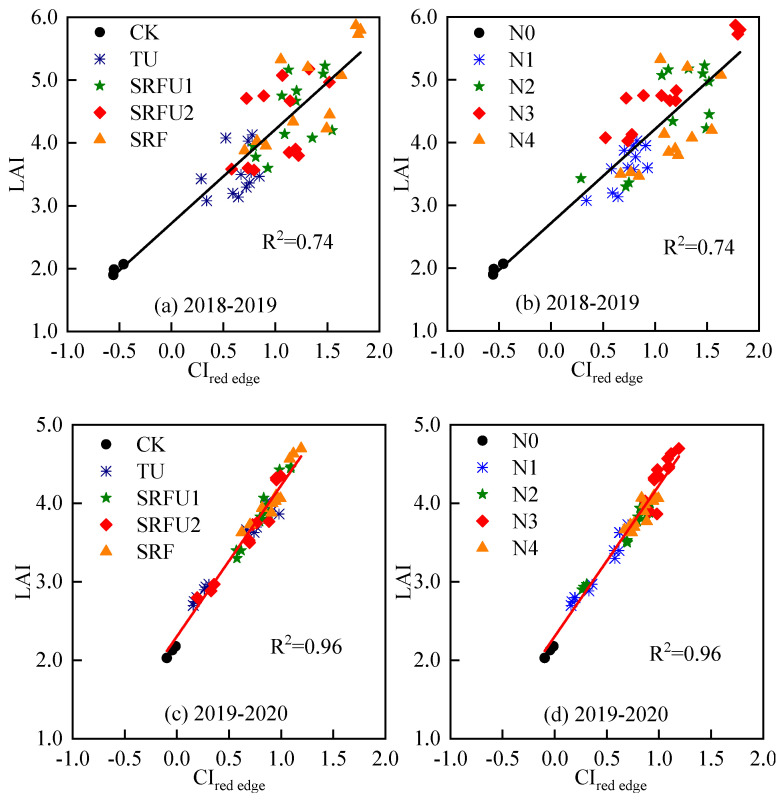
The relationship of LAI and CI_red edge_ of winter wheat under different nitrogen fertilizer types and nitrogen application rates in 2018–2019 (**a**,**b**) and 2019–2020 (**c**,**d**). TU is urea. SRFU1 and SRFU2 are urea blended with slow-release nitrogen fertilizer at ratios of 2:8 and 3:7, respectively. SRF is slow-release nitrogen fertilizer.

Figure 7 showed the relationships of LAI vs. CI_red edge_ for samples under different N fertilizer types and N application rates. The LAI and CI_red edge_ under different N fertilizer types and N application rates were significantly linearly correlated, with R^2^ were 0.74 and 0.96, respectively. CI_red edge_ was quite sensitive to winter wheat LAI variations regardless of N fertilizer types and N application rates (Figure 7 and Table 5). The algorithms for LAI estimated using CI_red edge_ for winter wheat under different N fertilizer types and N application rates were given in Table 5, which were LAI = 2.72 + 1.50 CI_red edge_ and LAI = 2.30 + 1.93 CI_red edge_ in 2018–2019 and 2019–2020, respectively.

### 3.4. Yield Estimation Based on CI_red edge_ of Winter Wheat

**Figure 8:**
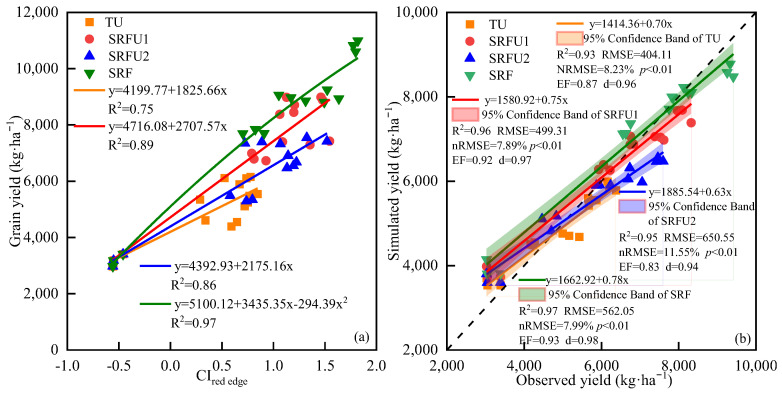
(**a**) The relationship of yield and CI_red edge_ of winter wheat under different nitrogen fertilizer types in 2018–2019. (**b**) Comparison of simulated and observed yield of winter wheat in 2019–2020. TU is urea. SRFU1 and SRFU2 are urea blended with slow-release nitrogen fertilizer at ratios of 2:8 and 3:7, respectively. SRF is slow-release nitrogen fertilizer.

The relationship between CI_red edge_ at the anthesis stage and grain yield after harvest of all samples in 2018–2019 winter wheat growing season was established. The algorithms for grain yield estimated using CI_red edge_ for winter wheat was significantly linearly correlated, which were Yield = 4083.95 + 3094.99 CI_red edge_ (R^2^ = 0.75) (Table 6). Regarding the various N fertilizer types, CI_red edge_ and grain yield were also significantly linearly correlated, and the statistical equations were Yield = 4199.77 + 1825.66 CI_red edge_ (R^2^ = 0.75), Yield = 4716.08 + 2707.57 CI_red edge_ (R^2^ = 0.89). Yield = 4392.93 + 2175.16 CI_red edge_ (R^2^ = 0.86) and Yield = 4965.00 + 3087.62 CI_red edge_ (R^2^ = 0.96) (Figure 8). Next, the accuracy of the algorithms model constructed by the CI_red edge_ collected from the UAV platform and grain yield in 2018–2019 was verified by the grain yield date samples in 2019–2020. The results showed that the accuracy of grain yield estimation by CI_red edge_ was relatively high (R^2^ = 0.75, RMSE = 730.88, nRMSE = 11.29%, EF = 0.78, d = 0.92). Regarding the various N fertilizer types, the accuracy of grain yield estimation by CI_red edge_ for data collected from UAV platform was improved in TU (R^2^ = 0.75, RMSE = 404.11, nRMSE = 8.23%, EF = 0.87, d = 0.96). However, the accuracy of grain yield estimation by CI_red edge_ for data collected from UAV platform significantly improved in SRFU1 (R^2^ = 0.89, RMSE = 499.31, nRMSE = 7.89%, EF = 0.92, d = 0.97), SRFU2 (R^2^ = 0.86, RMSE = 650.55, nRMSE = 11.55%, EF = 0.83, d = 0.94) and SRF (R^2^ = 0.96, RMSE = 562.05, nRMSE = 7.99%, EF = 0.93, d = 0.98) (Figure 8).

### 3.5. Construction and Comparison of Winter Wheat Grain Yield Prediction Models

**Figure 9:**
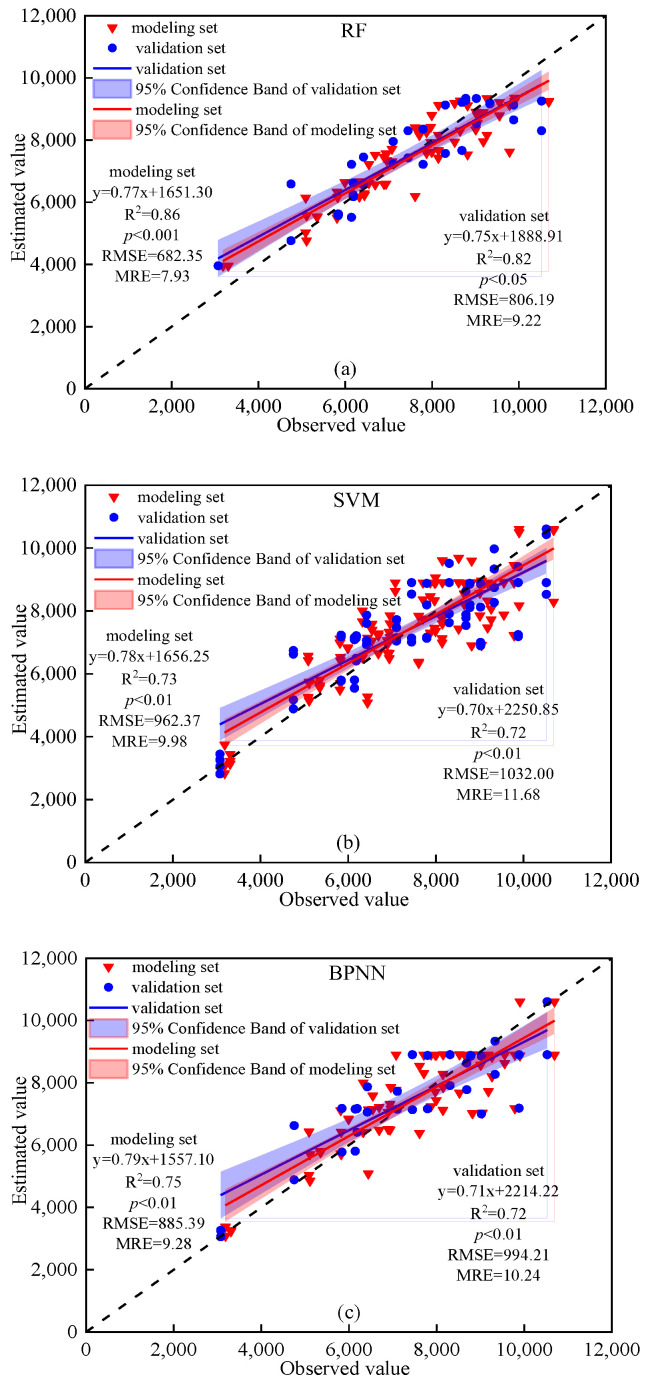
Grain yield prediction results of winter wheat by random forest regression models (RF), support vector machine model (SVM) and back propagation neural network model (BPNN) developed from CI_red edge_. (**a**) Grain yield prediction results of winter wheat by RF developed from CI_red edge_. (**b**) Grain yield prediction results of winter wheat by SVM developed from CI_red edge_. (**c**) Grain yield prediction results of winter wheat by BPNN developed from CI_red edge_.

The spectral index of CI_red edge_ was the independent variable, and the grain yield of maize was the response variable. Figure 9 shows the grain yield of winter wheat grain estimation results when using CI_red edge_ and by using the RF, SVM and BPNN techniques. The model accuracy was comprehensively evaluated from three aspects of R^2^, RMSE, and MRE. The results in Figure 9 demonstrate that the accuracy of CI_red edge_ based grain yield of winter wheat grain estimates vary among RF, SVM and BPNN, with validation R^2^: 0.72–0.86, RMSE: 682.35–1032.00 kg·ha^−1^, and MRE: 7.93–11.68, indicating that these models have good fitting accuracy and can be used to estimate winter wheat yield. Based on RF method, the R^2^ of winter wheat grain yield estimation model modeling and verification sets were 0.86 and 0. 82, RMSE was 682.35 and 806.19, and MRE was 7.93 and 9.22. Based on SVM method, the R^2^ of winter wheat grain yield estimation model modeling and verification sets were 0.73 and 0. 72, RMSE was 962.37 and 1032.00, and MRE was 9.98 and 11.68. Based on BPNN method, the R^2^ of winter wheat grain yield estimation model modeling and verification sets were 0.75 and 0. 72, RMSE was 885.39 and 994.21, and MRE was 9.28 and 10.24. For the same input variable and different modeling methods, the accuracy of the modeling and verification sets of the grain yield estimation model, constructed by the three modeling methods, were as follows: RF > BPNN > SVM, indicating that RF was the optimal model construction method. To summarize, the RF model was the best modeling method, which could extract the effective information of CI_red edge_ and grain yield of maze to a greater extent. To prevent overfitting in RF, SVM, and BPNN models, stratified *k*-fold cross-validation (k = 5), L2 regularization for SVM, maximum depth constraints for RF, and dropout layers for BPNN were implemented, consistently yielding validation R^2^ > 0.70 with <5% performance degradation between training and testing phases across all nitrogen treatment scenarios.

### 3.6. Comparison Between Winter Wheat Grain Yield Were Predicted Based on Spectral Index of CI_red edge_ and Measured Grain Yield

**Figure 10:**
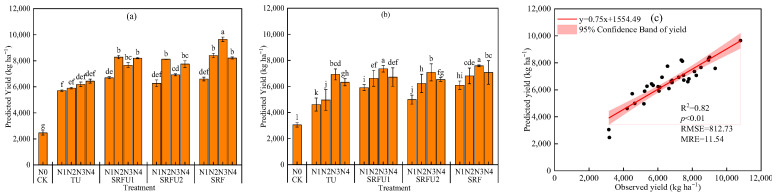
(**a**) Spectral index of CI_red edge_ prediction yield of winter wheat under different nitrogen fertilizer types and nitrogen application rates in 2018–2019. (**b**) Spectral index of CI_red edge_ prediction yield of winter wheat under different nitrogen fertilizer types and nitrogen application rates in 2019–2020. (**c**) The relationship between observed yield and predicted yield based on the data of the two-season experiment. TU is urea. SRFU1 and SRFU2 are urea blended with slow-release nitrogen fertilizer at ratios of 2:8 and 3:7, respectively. SRF is slow-release nitrogen fertilizer. Different letters indicate significance at the 5% level for the same year by the LSD test. Error bars are ±1 standard deviation of the mean (n = 3). The Y-bars on each data point should indicate SD.

Figure 10 shows the spectral index of CI_red edge_ prediction of grain yield and measured grain yield under different N fertilizer types and rates. Comparing the measured grain yield with the predicted grain yield, it was found that a positive linear relationship was established between the predicted grain yield and measured yield. The equations were y = 0.75x + 1554.49 (R^2^ = 0.82) (Figure 10). Regarding the various N fertilizer types, the predicted grain yield was consistent with the measured grain yield, and still followed the order of SRF > SRFU1 > SRFU2 > TU > CK. However, regarding the different N application rates, the grain yield was accurately predicted during the growing seasons of winter wheat grain in 2019–2020. In addition, comparing the predicted grain yield with the measured grain yield, it was found that there was a deviation when the grain yield was predicted by remote sensing. Samples with low grain yield values were overestimated by CI_red edge_ based grain yield estimates (Figure 10). On the contrary, samples with high grain yield values were underestimated by CI_red edge_ based grain yield estimates (Figure 10). In summary, the remote sensing prediction method based on the spectral index CI_red edge_ of the anthesis stage can effectively predict winter wheat grain yield. 

## 4. Discussion

### 4.1. The Relationship Between LAI and Grain Yield of Winter Wheat Under Different N Fertilizer Types and Rates

N is an essential element for regulating crop growth and development, playing an irreplaceable role in crop physiological function and overall metabolism, and is a key agricultural input to increase yield [11,44]. Changes in soil available N content can cause changes in crop yield, so early prediction of winter wheat yield is crucial for N management measures and food security in the Guanzhong Plain [7]. N not only affects the cumulative growth of dry matter, but also the expansion of leaf blades [14]. The reasonable application of N fertilizer can prolong green leaf duration and increase population photosynthetic time [45,46]. In this study, SRF and SRFU as optimized N fertilizers can effectively alleviate the adverse effects of fertilizer deficiency on winter wheat by delaying leaf senescence, increasing LAI, improving chlorophyll content and photosynthetic performance. This is consistent with the findings of Fan et al. (2021) [47] and Ma et al. (2021) [48], who reported that SRF and SRFU maintained higher leaf activity in plant canopy compared with urea, which is an effective way to increase dry matter accumulation and yield. Previous studies have shown that LAI, chlorophyll content and N content in crop canopy are used as yield predictors [19,49]. This may be due to crops regulating growth and development by adapting to the absorbed light profile, which is closely related to leaf growth and physiological characteristics, implying that the leaf area available for light interception could determine plant yield [50,51]. LAI is one of the most crucial variables in the growth model, and an important parameter reflecting canopy photosynthesis, respiration and transpiration, which is closely related to crop dry matter accumulation and yield formation [52]. Moreover, LAI has important parameters reflecting the nutritional status of crops, and monitoring LAI can thus provide vital references for field fertilization management, crop growth performance and yield estimation [15]. Related research in wheat and rice has proved the feasibility of yield estimation based on LAI [53,54].

The relationship between LAI and crop yield was not consistent at each growth stage [55]. This study analyzed the yield of winter wheat was estimated by LAI at various growth stages, and the results showed that the best timing and more accurate model for winter wheat yield estimation was found at the anthesis stage (*r* = 0.96 in 2018–2019 and *r* = 0.83 in 2019–2020). This may be due to the low correlation between early crop growth stage and grain yield because the demand for nutrients at the early stage of wheat is low, and the difference in crop growth under different N fertilizer treatments is not significant, so the early growth stage has less effect on yield [7,19]. In addition, wheat panicles become larger and yellower from the early stage of grain filling to the maturity stage, and the leaves and panicles will affect the canopy reflectance of wheat, thus affecting the accuracy of yield estimation, especially after the early stage of grain filling [19,56]. The relationship between canopy spectral reflectance and grain yield weakened during the filling stage and maturity stage as leaves senesce, chlorophyll content gradually decline, and assimilates are transferred to the grain, and the continuous development of reproductive organs will also cover the absorption and utilization of light by the canopy [5]. However, the anthesis stage is an important period for wheat to change from vegetative growth to reproductive growth. During this period, the growth and physiological indexes of wheat reach the peak state, and the highest light interception ability and utilization efficiency of leaves are beneficial to the formation of grains and play a decisive role in high yield of wheat [19,57]. Therefore, the anthesis stage is important in determining the number of grains and grain weight. In addition, the model of LAI and yield of winter wheat at the anthesis stage was established in this study. The results showed that both the positive linear relationship and the quadratic non-linear relationship can fit well the relationship between LAI and yield (R^2^ ≥ 0.94) (Table 4 and Figure 3). Moreover, our results also highlighted that the application of different N fertilizer types can significantly affect the relationship between LAI and yield. The correlation coefficient (R^2^) between yield and LAI of maize under SRF and SRFU was higher than that under TU. These results are due to the fact that SRF and SRFU increase the LAI during the vegetative growth stage of the crop compared with TU, which improved leaf growth, tissue carbohydrate accumulation and translocation to grains, thus increasing yield [18].

### 4.2. Response of Vegetation Indexes Calculated from UAV-Based Images to LAI During Anthesis Stage Under Different N Application Types and Amounts

Recently, remote sensing techniques are increasingly being used in agronomy management, especially irrigation and fertilization management [19]. In this study, SRF and SRFU were used as optimized N fertilizer management practices can effectively improve the growth physiology, increase crop yield and N use efficiency of winter wheat. Moreover, this study focuses on real-time monitoring of crop leaf growth by remote sensing, which was very important for assessing the impact of fertilization strategies on the growth of winter wheat and estimating the potential of winter wheat yield under different N types and N application rates. Previous studies have also confirmed the advantages of integrating canopy VI and canopy structure information extracted from UAV-based multi-spectral images in predicting yield [20]. Martins et al. (2023) reported that vegetation indices extracted from multi-spectral data can effectively describe the characteristics of crop growth [58]. The changes in LAI on the field canopy profile can be monitored using characteristic spectral signatures by remote sensing methods [59]. LAI provides the necessary link between remote sensing observation data and canopy state variables used as predictors of crop yield. Previous studies have predicted tomato yield through the correlation between LAI and vegetation index [60]. Zhou et al. (2017) used the single growth stage vegetation index (VIs) and multi-temporal generations from UAV multi-spectral, digital photos and LAI to estimate rice crop yield [27]. The results of this study showed that the six vegetation indexes (NDVI, OSAVI, EVI2, CI_red edge_, MTCI and NDRE) of winter wheat canopy were significantly correlated with LAI (R^2^ ≥ 0.72, Figure 5). Previous studies also have shown that VIs have a good correlation with LAI [61,62]. Liu et al. (2017b) also indicated that VIs and LAI showed a similar trend [63]. This was because the green and red edge have a wide range of spectral regions, thus their reflectance is the most suitable for non-destructive estimation of leaf surface area index. Furthermore, remote sensing data store information in multiple spectral bands, providing rich information required for crop yield estimation [64]. Previous studies only used remote sensing data or combined it with other data to predict crop yield [65]. This also indicated that the spectral and morphological characteristics of crop canopy were highly correlated, both of which can reflect the growth and development of crops and were directly related to its potential yield.

In this study, the prediction accuracy of six commonly used VIs as coupling indices was compared, and CI_red edge_ was the best coupling index. Conventionally, greenness indices extracted from NIR (Near-infrared) data, such as NDVI and EVI, were widely used to predict yield [66]. However, the utilization of potential in the region of the spectrum in red edge (i.e., 650–750 nm) has resulted in a significant recent advancement [19]. Combining the CI_red edge_ obtained from high spatial resolution UAV images with the LAI at the anthesis stage can significantly improve the LAI prediction accuracy of winter wheat compared with other vegetation indices, indicating that VI and LAI had internal correlation. Therefore, it is easier to obtain more accurate results by assimilating the vegetation index of CI_red edge_ to optimize LAI. These results were in line with the findings of Morier et al. (2015), who reported that CI_red edge_ has been recognized as a good linear estimator of canopy N content in both grassland and potato cropping systems [67]. These results, probably due to CI_red edge_, can reduce the influence of soil and non-leaf organs on reflectance during the growth stage of wheat to a certain extent, thus highlighting vegetation characteristics and improving prediction accuracy [19]. In addition, the noise equivalent values (NEΔLAI) of six VIs were calculated, and the dynamic response of NEΔLAI to LAI changes was analyzed (Figure 6) in this study. This parameter is helpful to understand the response of VIs to LAI. The results of this study showed that the NE△LAI behavior of CI_red edge_ was completely different from other vegetation indices, and it remained almost unchanged throughout the leaf area variation range. These results suggested that compared with the other five vegetation indices, the vegetation of CI_red edge_ could more accurately and stably predict the change in winter wheat LAI. However, when monitoring LAI based on the UAV platform, the mixed pixels of remote sensing images may lead to deviations in the expression of winter wheat growth status, thus limiting the ability to predict yield [68]. Previous studies have also shown that using different VIs as assimilation parameters will have a significant impact on crop growth and yield prediction accuracy [69]. Therefore, the use of vegetation indices obtained from remote sensing images to assessed crop growth status and estimated yield needs further study.

In this study, the canopy spectral characteristics of winter wheat at anthesis stage were still used to estimate the yield. Firstly, this is because the above results showed that the best period for yield estimation using LAI was the anthesis stage. Secondly, the reason for these results was that the canopy structure of the crop has been changing during the growth process, and the physiological and morphological changes in crop leaves during leaf expansion and maturation affect spectral receptivity and plant spectral characteristics [55]. Crop growth is very slow during the early stages and the leaves were not fully unfolded, so the estimation of LAI by remote sensing would be biased. In addition, the canopy could not completely cover the soil, and remote sensing would be affected by the soil background value when capturing canopy information [19]. The relationship between canopy spectral reflectance and grain yield weakened at the later growth stages as leaves senesced, LAI gradually declined, and assimilates were transferred to the grain [25]. Furthermore, the continuous development of reproductive organs will also cover the canopy characteristics derived from UAV-based spectral remote sensing [25]. Therefore, the prediction results of the filling stage and maturity stage data were also unsatisfactory (R^2^ = 0.65). In this study, the anthesis stage was the best prediction period for yield prediction using CI_red edge_ (R^2^ ≥ 0.75, Figure 8). Using the yield and CI_red edge_ model established in 2018–2020, the simulated yield of winter wheat in 2019–2020 was compared with the observed yield, and it was found that R^2^ ≥ 0.93. This may be due to the fact that crop canopy reflectance is less affected by the background soil, and the reflectance saturation phenomenon has not yet appeared at the anthesis stage. However, previous studies have shown that the accuracy of remote sensing data for yield prediction in winter wheat planting areas is increasing from seeding stage to maturity stage [70]. These results may be mainly concentrated in stem and leaf vegetables, that is to say, the canopy reflectance in the middle and late stages of crop growth was not affected by reproductive organs. In conclusion, although the potential of UAV-based multi-temporal image data integration for grain yield assessment has been confirmed, the prediction of grain yield based on UAV for different agronomic measures, different crops and different growth stages still needs further research.

### 4.3. Prediction of Winter Wheat Grain Yield Using Vegetation Index Based on Different Mechanical Learning Methods

In the field of remote sensing and agriculture, deep learning has successfully incorporated multi-variate and multi-temporal data [65]. This method has been widely applied to crop classification and yield prediction, achieving high accuracy and reducing the need for ground observations [55]. Recently, deep learning algorithms, which are advanced statistical models, such as random forest (RF), support vector machine (SVM) and back propagation neural network (BPNN), have become the preferred methods for crop yield estimation [71]. In this study, UAV-based multi-spectral images were collected at the whole growing period of wheat. Deep learning models, namely RF, SVM and BPNN, were used to combine different vegetation indices with wheat canopy information under different N fertilizer types. The results of this study showed that the deep learning model combining CI_red edge_ (optimal VI) and canopy information of LAI showed good potential in yield estimation, which was consistent with some previous research results. Chen et al. (2022) also reported that the method of predicting fruit-tree yield based on spectral and morphological features uses machine learning regression models to establish the relationship between independent variables and yield, so it has the advantage of not relying on the unique parameters of individual crops to predict yield [72]. Additionally, the results of this study also showed that deep learning technology can effectively capture the impact of CI_red edge_ changes based on UAV remote sensing on yield prediction [23]. When CI_red edge_ was used to predict yields, the simulated values were lower than those observed under high yield conditions and higher than those observed under low yield conditions.

RF, SVM and BPNN were three kinds of deep learning models commonly used in precision agriculture and remote sensing data analysis. The simulation results of RF, SVM and BPNN models in Figure 9 of this study also showed that the R^2^, RMSE and MRE of the modeling set and the validation set of the yield estimation model were greater than 0.73, 682.35, 7.93 and 0.72, 806.19, 9.22, indicating that these models have good fitting accuracy and can be used to estimate the yield of winter wheat. In addition, the accuracy of RF, SVM and BPNN in predicting yield was compared through a series of experiment data in this study, and it was found that RF (R^2^ = 0.86, RMSE = 682.35, MRE = 7.93 in modeling set and R^2^ = 0.82, RMSE = 606.19, MRE = 9.22 in validation set) performs better than SVM (R^2^ = 0.73, RMSE = 962.37 MRE = 9.98 in modeling set and R^2^ = 0.72, RMSE = 1032.00 MRE = 11.68 in validation set) and BPNN (R^2^ = 0.75, RMSE = 885.39, MRE = 9.28 in modeling set and R^2^ = 0.7, RMSE = 994.21, MRE = 10.24 in validation set) in evaluating and predicting yield accuracy. This is consistent with the findings of Li et al. (2022), who also reported that random forest (RF) can predict crop yield and was superior to multiple linear regression in all comparative statistical performance [73].

This study analyzed the measured yield and predicted yield under different N application rates and N fertilizer types, and found a positive linear relationship between them (R^2^ = 0.82), indicating that remote sensing technology can accurately predict winter wheat yield. Previous studies have shown that remote sensing data provide a solid basis for monitoring crop health, early detection of disease and estimation of yield, thereby increasing crop yield [66]. Yang et al. (2022) also reported that identifying the optimal canopy growth stage of winter wheat, yield prediction time, and observation frequency was crucial for improving the accuracy of yield prediction based on UAV remote sensing with lower time and labor costs [25]. Therefore, this study combined wheat canopy spectral information, LAI status and yield prediction results to accurately and timely adjust farmland water and fertilizer management measures, which is very important to maximize wheat yield. Moreover, the canopy information of winter wheat was usually affected by farmland water and fertilizer management measures and CI_red edge_ at the anthesis stage was the best index and period for estimating yield, thus CI_red edge_ index at the anthesis stage is recommended to predict wheat yield in this study. However, the study by Zhou et al. (2017) showed that the correlation between multi-temporal spectral VIs and grain yield was higher than that of single-phase VI because multi-temporal observation can provide rich crop growth information, thus alleviating the biases within mono-temporal observation [27].

UAV remote sensing technology has been widely used in agricultural production. From field data collection to guiding field management, it can be completed in a few hours, but there are still many problems worthy of attention in the application of future research. The limitations of this study include focusing on specific growth environments and crop varieties, which may limit the generalization ability of the model across different regions and wheat varieties. In addition, the interaction between key environmental variables (such as meteorological factors and soil characteristics) and complex texture features was not fully integrated, which may compromise the prediction accuracy. In the future, we will focus on incorporating key environmental variables, such as meteorological factors, soil characteristics and canopy structure into the model, which will help to establish a more robust estimation framework and further enhance the applicability of the model across diverse regions and crop varieties. The introduction of high-dimensional and multi-level texture feature combination strategy will also provide strong support for improving the prediction accuracy of the model. Moreover, using UAV remote sensing to monitor crop canopy information and predict yield, the data acquisition phase serves as the critical foundation for ensuring precision. In the process of data acquisition, hardware, environmental conditions and data acquisition time will affect the comparability and effectiveness of data. At present, the accuracy of data and the precision of the model can be improved by means of instrument calibration, selection of time flight with stable light and small wind speed, and establishment of the standardized data acquisition process. Therefore, future research on crop yield prediction based on spectral information of the UAV remote sensing platform needs to be carried out from more aspects and perspectives.

## 5. Conclusions

There was a significant correlation (R^2^ ≥ 0.85) between LAI and yield at four growth stages of winter wheat under different N application rates and N fertilizer types. The results showed that LAI at the anthesis stage exhibited a robust correlation with winter wheat yield, establishing its strong predictive potential for yield forecasting using LAI-derived metrics during this critical growth phase. By modeling the relationships between LAI and six vegetation indexes derived from remote sensing images, CI_red edge_ was identified as the optimal vegetation index for winter wheat yield prediction. The yield of winter wheat was estimated and verified by CI_red edge_. A highly significant positive correlation was demonstrated between observed and simulated yields under different N fertilizer types. The results of this study showed that RF performed better than SVM and BPNN in evaluating the accuracy of predicted yield. This study recommends that the suitable N application rate for winter wheat is 220 kg ha^−1^, and the optimized N application type is SRF. However, this study’s exclusive focus on the CI_red edge_ index necessitates further evaluation of yield simulation accuracy. Future research should integrate multi-spectral vegetation indices and key environmental variables to construct a more robust estimation framework.

## Figures and Tables

**Table 1:** Indices calculated from UAV-based images in this study.

Published Index	Reference
Normalized Difference Vegetation Index (NDVI) = (NIR − Red)/(NIR + Red)	(Rouse et al., 1974) [37]
Optimized Soil Adjusted Vegetation Index (OSAVI) = (1 + 0.16) × (NIR − Red)/(NIR + Red + 0.16)	(Rondeaux et al., 1996) [38]
(Enhanced Vegetation Index) EVI2 = 2.5 × (NIR − Red)/(NIR + 2.4 × Red + 1)	(Jiang et al., 2008) [39]
(Red-Edge Vegetation Index) CI_red edge_ = NIR/R_red edge_ − 1	(Gitelson et al., 2003) [40]
(MERIS Terrestrial Chlorophyll Index) MTCI = (NIR − R_red edge_)/(NIR + Red)	(Dash and Curran et al., 2004) [41]
(Normalized Difference Red-Edge Index) NDRE = (NIR − R_red edge_)/(NIR + R_red edge_)	(Gitelson and Merzlyak et al., 1997) [42]

NIR is the vegetation reflectance in the near-infrared band (790–810 nm). Red: red band at 670 nm and 690 nm wavelengths. R_red edge_: red-edge band at 710 nm and 730 nm wavelengths.

**Table 2:** LAI of winter wheat (*Triticum aestivum* L.) under different nitrogen fertilizer types and nitrogen application rates in 2018–2019 and 2019–2020.

Year	Treatment		Regreening Stage	Jointing Stage	Anthesis Stage	Filling Stage
2018–2019	CK	N0	0.56 i	0.67 j	0.99 k	0.37 i
	TU	N1	1.97 efgh	3.27 defg	3.14 j	1.57 h
		N2	3.42 a	3.56 bcde	3.37 ij	2.05 g
		N3	2.46 bcd	4.03 a	4.08 ef	2.74 e
		N4	2.31 cde	3.71 abc	3.50 i	2.54 ef
	SRFU1	N1	1.62 h	2.71 hi	3.78 gh	2.69 ef
		N2	2.11 defg	3.69 abc	5.17 b	4.08 a
		N3	2.48 bcd	3.36 cdefg	4.75 c	3.94 ab
		N4	2.35 cde	3.17 efg	4.14 de	3.67 bc
	SRFU2	N1	1.78 gh	2.47 i	3.59 hi	2.39 f
		N2	2.23 cdef	3.49 cdef	5.08 b	3.92 ab
		N3	2.52 bc	3.03 gh	4.71 c	3.37 cd
		N4	2.40 cd	2.64 i	3.85 fg	3.17 d
	SRF	N1	1.87 fgh	3.11 fg	3.96 efg	3.14 d
		N2	2.83 b	3.41 cdefg	4.34 d	3.43 cd
		N3	2.44 cd	3.90 ab	5.80 a	4.22 a
		N4	2.20 cdef	3.64 abcd	5.21 b	4.02 a
N fertilizer type (NT)			**	**	**	**
N application rate (NR)			**	**	**	**
NR × NT			**	**	**	**
2019–2020	CK	N0	0.67 h	2.32 l	2.68 h	2.13 k
	TU	N1	2.36 ef	2.53 jk	2.75 h	2.27 j
		N2	2.75 abcd	2.89 h	2.94 g	2.54 h
		N3	2.95 ab	4.23 a	4.27 c	3.88 b
		N4	2.77 abcd	3.49 d	3.67 e	3.21 e
	SRFU1	N1	1.94 g	2.44 k	2.90 g	2.30 ij
		N2	2.43 def	2.61 j	3.80 e	2.84 g
		N3	2.97 ab	4.08 b	4.45 b	3.90 b
		N4	2.82 abc	3.17 f	3.99 d	3.21 e
	SRFU2	N1	2.13 fg	2.29 l	2.89 g	2.22 j
		N2	2.38 ef	2.45 k	3.52 f	2.61 h
		N3	3.02 a	3.94 c	4.33 bc	3.76 c
		N4	2.88 abc	3.04 g	3.75 e	3.03 f
	SRF	N1	2.09 fg	2.48 k	3.03 g	2.38 i
		N2	2.54 cde	2.77 i	3.94 d	2.99 f
		N3	2.92 ab	4.18 ab	4.64 a	4.04 a
		N4	2.63 bcde	3.33 e	4.07 d	3.33 d
N fertilizer type (NT)			ns	**	**	**
N application rate (NR)			**	**	**	**
NR × NT			ns	ns	**	**

Notes: TU is urea. SRFU1 and SRFU2 are urea blended with slow-release nitrogen fertilizer at ratios of 2:8 and 3:7, respectively. SRF is slow-release nitrogen fertilizer. NT is N fertilizer type. NR is N application rate. Different letters within the column indicate the significance within the same year at the 5% level by the LSD test. ns represents not significant at *p* > 0.05. ** Significant at *p* < 0.01.

**Table 3:** Pearson correlation coefficient (r) and significance analysis of yield estimation using leaf area index at the regreening stage, jointing stage, anthesis stage and filling stage of winter wheat.

	2018–2019				2019–2020			
	RG	JO	AN	FI	RG	JO	AN	FI
Pearson correlation coefficient (r)	0.47	0.66	0.96	0.94	0.67	0.53	0.83	0.70
Significance analysis	**	**	**	**	**	**	**	**

Notes: RG: Regreening stage, JO: Jointing stage, AN: Anthesis stage, FI: Filling stage. ** Significant at *p* < 0.01.

**Table 4:** Yield estimation by LAI at the anthesis stage of winter wheat.

Year	N fertilizer Types	Fitting Function	R^2^
2018–2019	TU	Yield = 261.40 + 1455.88 LAI	0.95
	SRFU1	Yield = −1976.77 + 2901.34 LAI − 150.79 LAI^2^	0.99
	SRFU2	Yield = −1268.48 + 2547.84 LAI − 161.14 LAI^2^	0.97
	SRF	Yield = −3287.88 + 3762.64 LAI − 241.50 LAI^2^	0.97
	ALL	Yield = −827.30 + 1896.03 LAI	0.85
2019–2020	TU	Yield = −3588.13 + 4016.09 LAI − 390.40 LAI^2^	0.94
	SRFU1	Yield = −1338.63 + 2163.98 LAI	0.99
	SRFU2	Yield = −1116.09 + 2033.90 LAI	0.98
	SRF	Yield = −2134.29 + 2489.85 LAI	0.99
	ALL	Yield = −1858.49 + 2293.14 LAI	0.90

Notes: TU is urea. SRFU1 and SRFU2 are urea blended with slow-release nitrogen fertilizer at ratios of 2:8 and 3:7, respectively. SRF is slow-release nitrogen fertilizer.

**Table 5:** LAI estimation by CI_red edge_ of winter wheat under different nitrogen fertilizer types and nitrogen application rates.

Year	Fitting Function	R^2^
2018–2019	LAI = 2.72 + 1.50 CI_red edge_	0.74
2019–2020	LAI = 2.30 + 1.93 CI_red edge_	0.96

**Table 6:** Remote prediction of yield of winter wheat using CI_red edge_ from UAV images collected at the anthesis stage under different nitrogen fertilizer types.

N Fertilizer Types	Fitting Function	R^2^	RMSE	nRMSE	EF	d
TU	Yield = 499.77 + 1825.66 CI_red edge_	0.75	404.11	8.23%	0.87	0.96
SRFU1	Yield = 4716.08 + 2707.57 CI_red edge_	0.89	499.31	7.89%	0.92	0.97
SRFU2	Yield = 4392.93 + 2175.16 CI_red edge_	0.86	650.55	11.55%	0.83	0.94
SRF	Yield = 5100.12 + 3435.35CI_red edge_−294.39CI_red edge_^2^	0.97	562.05	7.99%	0.93	0.98
ALL	Yield = 4083.95 + 3094.99 CI_red edge_	0.75	730.88	11.29%	0.78	0.92

Notes: TU is urea. SRFU1 and SRFU2 are urea blended with slow-release nitrogen fertilizer at ratios of 2:8 and 3:7, respectively. SRF is slow-release nitrogen fertilizer. CI_red edge_ is Red Edge Vegetation Index. R^2^ coefficient of determination. RMSE is root mean square error. nRMSE is normalized root mean square error. EF is Nash–Sutcliffe model efficiency coefficient. d is index of agreement.

## Data Availability

The data that support the findings of this study are available on request from the corresponding author. The data are not publicly available due to privacy or ethical restrictions.

## Supplementary Materials

The following supporting information can be downloaded at: https://www.mdpi.com/article/10.3390/plants14131986/s1, Figure S1: Diagrams of architectures used in the experiments.
