# Supplementary figures and images for: UAV-Based Yield Prediction Based on LAI Estimation in Winter Wheat (Triticum aestivum L.) Under Different Nitrogen Fertilizer Types and Rates
Source: Plants (Basel). 2025 Jun 29;14(13):1986. doi: 10.3390/plants14131986 (PMC12252415; doi:10.3390/plants14131986)

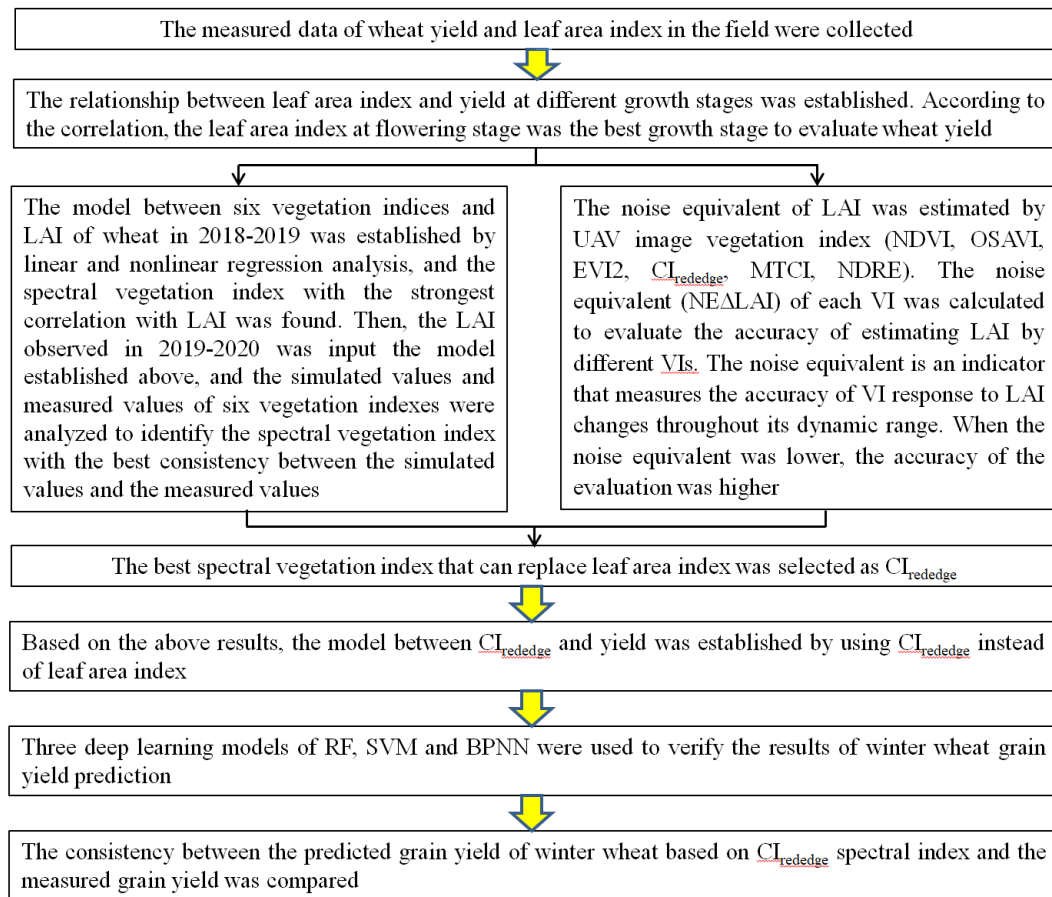

**Figure S1.** Diagrams of architectures used in the experiments

Supplement: Supplementary file 1 [file plants-14-01986-s001.zip › plants-3651898-supplementary.pdf]
